# A panorama of health inequalities in Brazil

**DOI:** 10.1186/s12939-016-0462-1

**Published:** 2016-11-17

**Authors:** Celia Landmann-Szwarcwald, James Macinko

**Affiliations:** 1 Laboratório de Informações em Saúde, Instituto de Comunicação e Informação Científica e Tecnológica em Saúde. Fundação Oswaldo Cruz, Rio de Janeiro, RJ Brasil; 2 Departments of Health Policy and Management and Community Health Sciences, UCLA Fielding School of Public Health, 650 Charles E. Young Dr. South, Room 31-235B, Center for Health Sciences, Los Angeles, CA 90095-1772 USA

Brazil is well known as a country with extremes in income and other social inequalities [1]. But in recent years, Brazil has made considerable strides in extending a range of social protections to the entire population. Notable accomplishments include achieving nearly universal health coverage, expanding community-based primary care and providing a robust conditional cash transfer program [2, 3].

This special edition of the *International Journal for Equity in Health* presents an overview of health inequities in contemporary Brazil. It provides a summary of progress made and identifies priority areas that will require additional efforts. The articles contained in the issue vary considerably in their methods and approach, but all make use of data from the most recent Brazilian National Health Survey or *Pesquisa Nacional de Saúde* (PNS 2013).

Based on strong evidence of the association between an individual’s (and even a society’s) social and economic circumstances and their health, monitoring health inequalities has become an essential feature of measuring national health progress and development. Such monitoring has shown not only that health inequalities are present in nearly every nation, but that their magnitude represents a social gradient that extends from the most to the least privileged in society. This implies that policies and programs must be assessed not only in terms of changes they may make in the aggregate, but also to the extent to which they reduce social inequalities among different population groups. Given the many types of health outcomes and the many factors by which different populations can be compared, such monitoring and evaluation is a daunting task that must be based on relevant, reliable, and frequent data collection and analysis.

National health surveys are essential tools to provide reliable information that can guide health and social services to better meet the needs and expectations of the population--whether in the area of health services, in health promotion and prevention, or in other social areas that affect health, longevity, and quality of life. In Brazil, the process of development of the PNS started in 2009 with the objective of responding to needs to evaluate national health policies and programs considered priorities by the Brazilian Ministry of Health. Planning for the survey, which was carried out in 2013 as a partnership between the Oswaldo Cruz Foundation (Fiocruz) and Brazilian Institute of Geography and Statistics (IBGE), involved data collection in three main areas: health status, health services (access, utilization, continuity of care, and health care financing), and surveillance of risk factors for illness and injuries. Equity, an explicit goal of the Brazilian national health system, also formed a priority area.

In terms of design, the 2013 PNS is a household-based survey representative of the Brazilian noninstitutionalized population at the national, regional, state, and major metropolitan area levels [4]. The PNS builds upon the IBGE master sampling frame selected by cluster sampling in three stages (census tracts, households, and individuals) with stratification of the primary sampling units. In the 2013 PNS, 64,348 households and 60,202 individuals were interviewed in person. Survey results, including all household, individual, and biomarker (anthropometrics, blood pressure) data are publicly available and can be accessed free of charge and without prior authorization from the following site: http://www.ibge.gov.br/home/estatistica/populacao/pns/2013.

Turning to the present issue, its 14 articles span the scope and breadth of health inequalities in Brazil. They touch on different ways of assessing population health and illness and employ different approaches to comparing such outcomes among different segments of the Brazilian population. Each of these articles gives a unique perspective on the magnitude and the complexity of health inequalities and their correlates in Brazil; together, they provide parts of a larger roadmap of progress made and challenges ahead. By presenting this panorama together in one issue it is hoped that it will reinforce calls to strengthen health and social programs within Brazil and also contribute to the larger global conversation and burgeoning social movement to prioritize actions that tackle the social determinants of health and well-being.

In terms of health status, the edition includes an article on regional disparities in healthy life expectancy by Szwarcwald et al. [5] and an article by Barros et al. [6] which assesses inequalities in several health behaviors associated with risk of chronic conditions.

Given the main epidemiologic challenges facing contemporary Brazil, a number of articles address chronic diseases. The article by Malta et al. [7] presents predictors of chronic disease prevalence and comorbidity. The article by Alves et al. [8] shows the complex interplay of education, gender, and race in measured hypertension, while the article by Beltran-Sanchez et al. [9] documents distinct trends in educational inequalities in chronic conditions over the past 15 years.

In terms of injuries, Morais Neto et al. [10] discuss important disparities in road traffic injuries by regional and individual characteristics, while Gattengo et al. [11] demonstrate trends in intimate partner violence against women following the passage of legal protections (Maria da Pena law) in 2006.

In relation to heath services and the performance of the Brazilian national health system, França et al. [12] document important reductions in inequalities in reproductive and maternal health interventions over the past 4 decades, while Mullachery et al. [13] show a slight deceleration in progress made in assuring equitable access to healthcare services since 2008. Boccolini et al. [14] identify characteristics of the more than 20 million people who still underutilize the Brazilian health system and Dourado et al. [15] demonstrate the importance of the Family Health Strategy in providing a usual source of healthcare as well as remaining regional disparities in its distribution. The article by Lopes et al. [16] highlights individual and regional factors associated with disparities in the prevalence of depression and in access to effective treatment and Theme-Filha et al. [17] document inequalities in preventive cancer screening for women, regional disparities and correlation with other health behaviors. Finally, Lima-Costa et al. [18] demonstrate a distinct gap in access to and use of needed informal and formal care for older Brazilians with functional limitations.

In closing, we wish to thank the IJEqH editors, Efrat Shadmi and Leiyu Shi for their encouragement; Ana Lorena Ruano and Pricila Mullachery for their incredible editorial support; the authors for preparing such interesting and important papers; our superlative guest commentators, Michael Marmot and Cesar Victora for their wisdom and insights; and the Brazilian Ministry of Health for its support of the PNS and this edition.
